# Public-on-private dual practice among physicians in public hospitals of Tigray National Regional State, North Ethiopia: perspectives of physicians, patients and managers

**DOI:** 10.1186/s12913-017-2701-6

**Published:** 2017-11-10

**Authors:** Goitom Gigar Abera, Yibeltal Kiflie Alemayehu, Jeph Henry

**Affiliations:** 1 Tigray Regional Health Bureau, Mekelle, Tigray Ethiopia; 2 Department of Health Services Management, College of Public Health and Medical Sciences, Jimma University, Jimma, Oromia Ethiopia; 3 Yale University School of Public Health, New Haven, CT USA

## Abstract

**Background:**

Physicians who work in the private sector while also holding a salaried job in a public hospital, known as “dual practice,” is one of the main retention strategies adopted by the government of Ethiopia. Dual practice was legally endorsed in Tigray National Regional State, Ethiopia in 2010. Therefore, the aim of this study was to explore the extent of dual practice, reasons why physicians engage in it, and its effects on public hospital services in this state in northern Ethiopia.

**Methods:**

A cross-sectional study using mixed methods was conducted from February to March 2011 in six geographically representative public hospitals of Tigray National Regional State. A semi-structured, self-administered questionnaire was distributed to all physicians working in the study hospitals, and an interviewer-administered, structured questionnaire was used to collect data from admitted patients. Focus group discussions were conducted with hospital governing boards. Quantitative and qualitative data were used in the analysis.

**Results:**

Data were collected from 31 physicians and 449 patients in the six study hospitals. Six focus group discussions were conducted. Twenty-eight (90.3%) of the physicians were engaged in dual practice to some extent: 16 (51.6%) owned private clinics outside the public hospital, 5 (16.1%) worked part-time in outside private clinics, and 7 (22.6%) worked in the private wing of public hospitals. Income supplementation was the primary reason for engaging in dual practice, as reported by 100% of the physicians. The positive effects of dual practice from both managers’ and physicians’ perspectives were physician retention in the public sector. Ninety-one patients (20.3%) had been referred from a private clinic immediately prior to their current admission-a circular diversion pattern. Eighteen (19.8%) of the diverted patients reported that health workers in the public hospitals diverted them.

**Conclusions:**

Circular diversion pattern of referral system is the key negative consequence of dual practice. Physicians and hospital managers agreed that health worker retention was the main positive consequence of dual practice upon the public sector, and banning dual practice would result in a major loss of senior physicians. The motive behind the circular diversion pattern described by patients should be studied further.

## Background

Physician dual practice refers to full-time salaried public sector medical doctors practicing simultaneously in the private, for-profit sector [1, 2]. Also termed “public-on-private,” “moonlighting,” or “multiple job holding,” physician dual practice is present in almost all countries [1–3], but the extent to which physicians engage in dual practice, their motives for doing so, the consequences of dual practice, and the regulatory options depend highly on the local context [1–5].

Public-on-private dual practice is often promoted as a means to supplement low government salary rates, thus encouraging physician retention in the public sector and increasing healthcare access, particularly in low- and middle-income countries (LMICs) [5–8]. It has also been argued to reduce public sector waiting times by stimulating additional effort from physicians with profit incentives [9]. Additionally, dual practice can allow for provision of additional and/or higher quality services that are excluded from the public health service bundle due to low demand or low cost-effectiveness [9].

On the other hand, dual practice has been criticized as reducing the quality of public sector services by incentivizing physicians to divert time, attention, and resources to their private practices [1, 2, 6, 9]. Physicians have also been accused of patient diversion either by direct referral or by more subtle means, like manipulating increased public sector waiting times in order to stimulate demand for their private services [1, 2, 6, 9]. In most LMICs, regulations to reduce the negative effects of dual practice are either completely lacking or poorly implemented because of low enforcement capacity [4].

In Ethiopia, a low-income country in sub-Saharan Africa, the public health sector has long been suffering from a shortage of medical doctors [10]. The number of physicians in the country working in the nation’s public hospitals suffered a sharp decline from 1658 in 1989 to only 638 in 2006 [10] and in Tigray region(in which this study was conducted) the number of physicians continued to decline from 84 in 2006 to 77 in 2010. Due to high physician attrition rates, rapid population growth, low production, and increased post-graduate enrollment, the country’s physician-to-population ratio of 1:36,158 is far below the World Health Organization (WHO) recommendation for developing countries of 1:10,000 [10, 11].

As is the case with physicians in many other low-income countries, the major reason Ethiopian physicians leave the public sector is believed to be unmet salary expectations coupled with higher earning potential in the domestic private sector or in the international market [10, 12]. Therefore, allowing dual practice is one of the major physician retention strategies of the Tigray government endorsed in 2010 in Tigray region [13]. Its job allocation system is yet another strategy for retaining new medical graduates. Most students’ education is funded by the government, and in exchange for free training, health workers are required to serve a fixed number of years in a randomly selected public facility (3 years for GPs, 4 years for specialists) before receiving their license. Only after completing their service obligation or paying a large fee are they “released” from the public sector with their credentials [12].

Tigray National Regional State (TNRS), the northernmost of Ethiopia’s nine regions, officially introduced private-on-public dual practice in 1993. The region faces a critically poor physician to population ratio of 1:58,000 with 77 physicians working in the public sector in 2010 [13]. After the introduction of DP this number steadily increased and doubled by 2015(153) [13]. There are 15 general hospitals and 1 referral hospital in the public sector, while there are 127 privately owned clinics (26 higher-level clinics, 35 medium clinics, and 66 small clinics). Physicians working at public hospitals own 26 (20.5%) of the region’s private health facilities.

The new health financing strategy introduced in 1998 increased governmental health expenditure and dramatically shifted the financing structure of public sector health facilities, enabling them to retain revenue collected from user fees in addition to the annually allocated governmental budget [13, 14]. Nevertheless, public health facilities still struggle with tight budgets as government spending on healthcare remains far below the average in the rest of Africa, and many patients qualify for fee-waivers or exemptions [15–17].

Strengthening the public-private partnership is stated as part of the Tigray Regional Health Bureau’s agenda [13]. Private facilities provide selected fee-exempt services (DOTS, HCT, ART, PMTCT, reproductive health services, malaria services, etc.) through service provision contracts with the government [13]. Public hospitals are also encouraged by the government to operate “private wing” services which use the public hospital facilities outside of working hours [11]. In the “private wing,” patients pay a fee higher than that applicable in the main public facility in order to avoid waiting, and a fixed proportion is transferred to the public facility [8].

According to routinely reported data, the average bed occupancy rate in Tigray’s public hospitals hovers around 47% [18], yet many patients complain of long waiting times and other difficulties accessing public sector services [12, 16]. A study conducted on health workers’ performance in Ethiopia reported that absenteeism of health workers, pilfering of resources, illicit charging, and diversion of patients to the private sector are major problems [12]. However, the role of dual practice among physicians is not well studied in the Ethiopian context. Therefore, the objectives of this study were (1) to assess the extent of dual practice among physicians, (2) describe the situation of DP in Tigray region/Ethiopia (3) to assess the positive and negative consequences of dual practice on public sector service from the perspectives of physicians, patients, and hospital managers in TNRS, Ethiopia.

## Methods

### Study area

The study was conducted in TNRS, northern Ethiopia; its capital city (Mekelle) located 783 km from Addis Ababa, capital city of Ethiopia.TNRS is one of the nine regional states of the Federal Democratic Republic of Ethiopia located in the northern part of the country. It is bordered by Eritrea to the north, Sudan to the west, Afar Region to the east and Amhara Region to the south. The total area of the region is about 54,569.25 km^2^ and the elevation ranges from 600 to 2700 m above sea level. There are 6 administrative zones including one special zone, Mekelle Zone(western zone, north west zone, central zone, eastern zone, south east zone, southern zone and Mekelle zone). The region has 52 *Woredas* (34 rural and 18 urban) and 814 *Kebeles* (753 rural and 61 urban). According to the 2007 EC census projection, the region has a total population of 5,055,999 (49.2% male and 50.8% female).

The region had 13 governmental hospitals of them 6 where zonal and 7 were districts. (Kahsay-abera hospital is found in the western zone, Sihul hospital in north west,St Mary hospital in central, Adigrat in eastern zone, Mekelle hospital in Mekelle zone and Lemlem-karl in southern zone) since the southeastern zone is found to adjacent to Mekelle has no zonal hospital. Among these hospitals there were 56 physicians, of which 42 worked in the zonal hospitals where we undertook our study.

### Study design and period

A cross-sectional study was conducted from February to March 2011 in six public hospitals in TNRS (Kahsay Abera Hospital, Shul Hospital,St Mary Hospital, Adigrat Hospital, Mekelle Hospital, and Lemlem Karl Hospital).

### Inclusion and exclusion criteria

All patients admitted for at least two days in the six study hospitals during the study period were included to this study whereas too ill patient and unable to respond verbal communication were excluded from the study. All physicians who gave informed consent and work in the study hospitals were included to this study.

### Sample size and technique

Purposive sampling was used to select geographically representative hospitals for inclusion in the study. These hospitals represent all parts of the region.

Since the number of patient admitted in the zonal hospital were small in number and manageable all admitted patients were surveyed using the consecutive sampling method. All physicians who work in the study hospitals during the study period were also included to the study.

### Data collection

A self-administered, semi-structured, pre-tested questionnaire in English was distributed to all physicians working in the study hospitals during the study period. Physicians were asked about their medical specialization, number of years served in the public sector, monthly public sector salary, and type of employment in the public sector using a structured questionnaire. They were also asked to describe their level of involvement in dual practice if any, their reasons for engaging in dual practice, their opinions regarding its effect on public hospital service provision, and their recommendations to improve physician retention by using open ended questions. For this study Dual practitioner was operationally defined in two categories including (1) a full-time salaried public sector medical doctors practicing simultaneously in the private, for-profit sector outside the public hospital (2) physicians who work in the private wing of a public hospital [private practice ‘within’ public practice facilities]. Completed questionnaires were sealed by the respondents and collected by the study supervisors.

Admitted patients were interviewed using a structured, pre-tested questionnaire translated into Tigrigna, the local language. Data were collected on patients’ socio-demographic characteristics, their referral history and prior visits to private clinics, and their opinions on dual practice. High school graduates trained on the data collection tools conducted the interviews.

At each study hospital, a focus group discussion (FGD) was held with six members of the management committee, excluding the medical directors to avoid bias or undue influence. The participants discussed their reflections on the positive and negative effects of dual practice on public hospital services.The FGD included the following questions: what positive side do you observe from dual practice; what drawbacks did you observe in your hospital related to dual practice; how do you manage when problems occurred associated with dual practice and what is your opinion if the government bans dual practice?

### Data analysis

Quantitative data were cleaned and entered, and frequencies and descriptive statistics were computed using SPSS Version 16.0. Audio recordings of the FGDs were transcribed to text, coded, and analyzed according to thematic areas. Qualitative data from the FGD and open ended questions were summarized into categories based on the common thematic area that respondents reply. Small sample size precluded formal content analyses; a few key responses are reported here for context. Data from the three sources were triangulated to determine the extent of dual practice, the reasons physicians engage in it, and its impacts on public sector services.

### Ethical approval and consent to participate

Prior to the study, ethical clearance was obtained from Ethical Review Committee of the College of Public Health and Medical Sciences of Jimma University. All study participants including hospital administrators, physicians and patients were informed about the purpose of the research and how responses will be reported. Confidentiality and anonymity were maintained.

## Results

### Physicians’ perspective

All of the physicians (*N* = 31) working in the six study hospitals during the study period completed and returned the self-administered questionnaire, for a 100% response rate. Fifteen (48.4%) were general practitioners (GPs) and 16 (51.6%) were specialists. Table 1 shows the characteristics of the respondent physicians by type (GP or specialist). All of the respondents were full-time permanent employees of government hospitals expected to work 39 h per week. Their average monthly government salary was 3465 Birr (147 USD and ranged from 1997 EtB to 6000 EtB.(124-375USD).

**Table 1:** Background characteristics of physicians at six Zonal hospitals in Tigray National Regional State, February 2011 by level

Variable	General Practitioner (*N* = 15)		Specialist (*N* = 16)		All Physicians (*N* = 31)	
Age in years (Mean ± SD)	27.0 ± 4.07		39.1 ± 3.48		33.3 ± 7.19	
Gender						
Male	13	86.7%	16	100%	29	93.5%
Female	2	13.3%	0	0%	2	6.5%
Marital status						
Married	0	0%	13	81.2%	13	41.9%
Single	15	100%	3	18.8%	18	58.1%
Public sector service years (Mean ± SD)	2.3 ± 2.76		13.9 ± 4.39		8.3 ± 6.15	
Monthly public sector salary in Ethiopian Birr (Mean ± SD)	2436.00 ± 180.20		4430.00 ± 306.40		3465.00 ± 996.40	
Private Sector Involvement						
Owns a Higher Clinic	1	6.7%	11	68.8%	12	38.7%
Owns a Medium Clinic	2	13.3%	2	12.5%	4	12.9%
Works part-time in a private clinic	2	13.3%	3	18.8%	5	16.1%
Works in private wing of public hospital	7	46.7%	0	0.0%	7	22.6%
None	3	6.7%	0	0.0%	3	9.7%

All but three of the physicians (*N* = 28, 90.3%) were dual practitioners, engaging in the private sector to some extent with 12 (38.7%) reporting that they own private higher clinics, 4 (12.9%) owning medium clinics, 5 (16.1%) practicing as part-time employees of private clinics owned by others, and 7 (22.6%) working in the private wing of a public hospital. All of the 16 specialist physicians were dual practitioners. The three physicians working solely in the public sector were all GPs with less than two years of experience. The 16 physicians who owned their own private clinics, including 13 specialist doctors, reported spending an average of 4 h and 10 min and consulting an average of 19 patients per day at their private practice. The mean service duration was 8.3 years among all physicians, and 12.8 years among the 16 physicians who owned their own private clinics. The maximum service duration in the public sector was 22 years.

The major reason public sector physicians reported engaging in private practice, as stated by all 28 dual practitioners, was to earn additional income. Dual practitioners also mentioned improving their skills (*N* = 4, 14.3%) and creating access for the community (*N* = 4, 14.3%) as reasons for engaging in dual practice. When describing their motivations for staying in the public sector, dual practitioners most commonly cited government-provided incentives such as top-up payments (*N* = 19, 67.9%), duty allowances (*N* = 16, 57.14%), and housing allowances (*N* = 10, 35.7%). One specialist mentioned a moral obligation to serve the people and the country, while another revealed that the opportunities for patient diversion was a motivation for staying in the public sector:

*“We are working to promote our self in [public] hospitals to divert directly or indirectly the patients to our private clinics” —*A dual practitioner

Most (*N* = 22, 70.96%) physicians believed that dual practice has a positive consequence on public sector services, primarily with regard to physician retention, sharing the high patient load of public facilities, and concentrating public hospital resources on the most needy patients. From the 16 specialist physicians, the majority (*N* = 12, 75.0%) said they would leave their job in the public sector if the government banned dual practice, 2 (12.5%) were unsure how they would react, and 2 (12.5%) would stop their private practice and continue with their public hospital employment. Among the 12 dual practitioner GPs, the majority (*N* = 9, 75.0%) said they were unsure how they would react. In general, physicians felt very strongly about dual practice as a retention strategy.

*“Banning private practice would be a big mistake and the community, which the government thinks will benefit, will not.”* —A dual practitioner

*“It should be encouraged, as the salary of physicians is very much low. Working in the private clinic will improve their income and make them stay in the facility, and this will increase the service given in general, both in the public and private sector”*— A dual practitioner

Physicians mentioned early departure (*N* = 13, 41.9%), late arrival (*N* = 8, 25.8%), reduced attention to their public hospital job (*N* = 7, 22.6%), taking long breaks due to tiredness (*N* = 5, 16.1%), and absenteeism (*N* = 3, 9.7%) as some of the negative consequence of dual practice on public sector services. However, many physicians also noted that poor organization and management in the public hospitals also contributes to lack of motivation and absenteeism during the public working hours.

*“Poor behavior of physicians depends on the personal behavior of the physician and the strength of the management body of the hospital. It doesn't become influenced whether he/she works in a private clinic.”* —A dual practitioner

Accordingly, 20 (64.5%) physicians recommended improving the public hospitals’ working environment in order retain physicians (e.g., increasing availability of necessary medical supplies and instruments, decreasing bureaucracy, improving hospital management).

### Patients’ perspective

A total of 449 patients who were admitted to the study hospitals for at least two days prior to the interview participated in the study. Table 2 shows their socio-demographic characteristics. Only 153 (34.1%) patients had ever visited private clinics in the past. Half of the interviewed patients (*N* = 222, 49.4%) felt that it is good to allow physicians to hold both private and public jobs, 193 (43%) felt it is bad, and 34 (7.6%) had no opinion.

**Table 2:** Characteristics of admitted patients at six Zonal hospitals, Tigray National Regional State, February 2011

Characteristics	Frequency (*N* = 449)	Percent
Average age of respondent	37 years	
Gender		
Male	266	59.2%
Female	183	40.8%
Educational status		
Illiterate	214	47.7%
Can read and write	109	24.3%
Elementary or secondary	105	23.4%
College and above	21	4.7%
Marital status		
Married	310	69.0%
Single	95	21.2%
Divorced	36	8.0%
Widowed/er	8	1.8%
Ever visited private clinics		
Yes	153	34.1%
No	296	65.9%

From the 449 patients, 91 (20.3%) had been referred from private clinics owned by dual practitioners immediately prior to their current hospital admission. These 91 patients were asked about the advantages of private clinics, and their responses are shown in Table 3. The most commonly stated advantages of private clinics were better care and treatment (75.8%) and shorter waiting times (65.9%), while many patients expected some kind of preferential treatment at the public hospital based on their prior visit to a private clinic (Table 3). This expectation for preferential treatment was not unfounded: all of the 91 patients reported being initially denied services at the public hospital when following the normal public sector referral pattern but eventually receiving the service at the public hospital after being referred from a private clinic. Patients reported experiencing these circular referral patterns when trying to access radiologic investigations such as X-ray and ultrasounds (76.9%), hospital beds (63.7%), and surgical services (28.6%). Health professionals and physicians were reportedly responsible for diverting 18 of the 91 patients (19.8%) to the private clinics, from which they were finally referred back to the public hospitals (Table 4).

**Table 3:** Advantages of private clinics from the patients’ perspective, Tigray National Regional State, February 2011

Factor	Frequency (*N* = 91)	Percent
Better care and treatment in private sector	69	75.8%
Shorter waiting times in private sector	60	65.9%
Better attention in public hospitals after referral from private clinic	56	61.5%
Easier access to a bed in public hospital after referral from private clinic	36	39.6%

**Table 4:** Diversion to private clinics among inpatients in six Zonal hospitals in Tigray National Regional State, March 2011

Who diverted you to private clinic?	Frequency (*N* = 91)	Percent
Self	41	45.1%
Family	24	26.4%
Health workers and physicians	18	19.8%
Other	7	7.7%

### Hospital management’s perspective

Focus group discussions were conducted at the six study hospitals, with six management committee members participating in each. Participants strongly believed that the main positive impact of the current dual practice policy is retention of specialist clinicians in public hospitals. Most discussants considered attrition of such physicians to be automatic if dual practice were to be banned, nearly equating the decision to allow or ban dual practice with the decision to have or not have physicians in hospitals. Responding to a question about the advantage of allowing dual practice in hospitals, one discussant said:

*“…The presence of senior physicians enables our hospital to manage difficult cases and improves the quality of care provided to patients …” —*CEO of a study Hospital

FGD participants also identified patient load sharing between public and private health facilities as a positive impact of dual practice on public health services.

Hospital management members identified early departure and late arrival as the most frequently observed negative consequences of dual practice on hospital services. In addition, denial of available hospital services and associated abuse of public resources, patient diversion, and delayed response of on-call physicians to emergencies were identified as major problems.

## Discussion

In this study, we hypothesized that the extent of dual practice is increased and this had a positive effect in retaining physicians and this would also associated with negative emerging effects. Therefore, it was obtained that nearly all physicians practice DP and this was associated with decreasing waiting time and circular diversion pattern of patients.

### Extent of dual practice

In this study, dual practice was nearly universal among public sector physicians in TNRS. In comparison, a non-representative study in Portuguese-speaking African countries found 63.2% of public sector physicians were engaged in dual practice [7] versus 61.6% in the capital cities of Cape Verde, Guinea Bissau, and Mozambique. [8]. All senior physicians (specialists) were engaged in dual practice in our study. This is higher than reported by a study conducted in Peru, which showed that 72% of specialists were dual practitioners [19]. The higher level of dual practice in our study may be due to the recent legalization of dual practice in the region and the positive government position, relatively lower public sector salaries of physicians, governmental job allocation system, differences in regulatory capacity and mechanisms, and variation in the market for health services.

### Reasons physicians engage in dual practice

A common assumption is that public sector physicians who engage in dual practice are motivated by self-interest and compromise their commitment to patients in pursuit of financial gain. Similarly to other studies in LMICs [1, 20], this study found that the main reason physicians at public hospitals engage in dual practice is to obtain a better income when the government salary is too low. Other studies, including one conducted in Ethiopia, have also reported non-financial factors such as higher resource availability, clinical autonomy, and managerial efficiency as reasons why physicians prefer working in private facilities [12, 19, 21]. This suggests that increasing government salary of physicians in public hospitals might de-incentivize engagement in dual practice and thus reduce any negative effects on public hospitals. Nevertheless, these findings should be interpreted cautiously, considering the small sample size and short study period.

In light of extremely low government salaries, it is surprising that any physicians remain as civil servants, assuming that profit maximization is the main underlying motive for dual practice [1]. In this study, most physicians stated that government incentives like top-up, duty allowance, and housing allowance motivated them to stay in their government job, while at the same time maintaining that these incentives should be increased. One physician also mentioned the opportunity to divert patients as an incentive to stay in the public hospital. Only a few physicians reported non-financial advantages of working in the public sector, but other studies have noted various non-financial reasons why dual practitioners stay in the public sector, such as opportunities for professional development training, increased contact with pathologically complex patients, academic activities like teaching and research, serving the poor, and security of the stable monthly salary and government benefits [12, 19] this implies that physicians in our study setting are primarily financially motivated and that the government can retain physicians with attractive incentive packages.

### Consequence of dual practice on public sector service

In this study, physicians and hospital managers largely agreed that the positive consequence of dual practice on public hospital services were physician retention and sharing of the patient load. The FGD participants also stated that the presence of senior doctors in public hospitals enables them to manage difficult cases and improves quality of care. This is in agreement with previous findings which found that dual practice contributes to an increase of quality in public sector health care through creating a competitive market for health [1, 6]. Most economic models and evidence in the literature agree that the opportunity for dual practice is highly essential for keeping skilled physicians in the public sector, especially in situations where the government cannot afford to pay competitive salaries [6]. Accordingly, 75% of specialists said they would leave the public sector if dual practice were banned, compared with 46% in Peru, an upper-middle income country [19]. Thus, without the ability to increase physicians’ salaries, hospital managers in TNRS are faced with accepting the detrimental effects of dual practice on service provision in order to retain their highly skilled staff. This suggests that banning private practice is inadvisable in our study setting, although it is being practiced in some countries like Canada, China, and in some states of India and Ghana [22–24]. Many other studies confirm that banning dual practice leads to migration of highly skilled physicians from public sector and worsens the quality and social welfare in public hospitals, especially in LMICs. Since the adoption of DP in Ethiopia in 2006 the number of public sector physicians almost doubled to 2016, suggesting that civil service retention. In addition, experience shows that bans are difficult to enforce, and that dual practice exists outside the regulatory jurisdiction of the government despite the presence of a legal ban [4, 8, 22, 23, 25, 26].

Both physicians and hospital managers agreed that resource outflow in the form of absenteeism from the public sector was a major problem. Patients in this study also confirmed limited access, long waiting times, and dissatisfaction with the public hospital services. Absenteeism has also been documented in several Latin American countries. For example, Venezuelan doctors missed 37% of their contracted service hours and in Costa Rica, 65% of doctors and 87% of nurses felt that physicians were unjustifiably absent from work [1]. Studies also showed that absenteeism are also common in sub-Saharan African countries [27] and Ethiopia [12]. Interestingly, this study did not report material resource outflow as a problem, but a study in Addis Ababa, Ethiopia reported that drug pilfering from public facilities for use in the private sector was a serious problem [12]. This implies that public hospitals’ policies may be too weak to control early departure and late arrival of physicians. Clear and strict rules to prevent these problems should be developed.

In this study, patient diversion from the public sector to private clinics was found to be a common practice acknowledged by physicians, hospital managers, and patients alike. According to the referral histories of 91 patients who were referred from private clinics immediately prior to their current admission at the public hospital, all experienced a circular diversion pattern. Patients were initially denied available services at the public hospital, only to receive the service at the public hospital after being referred from a private clinic. A qualitative study in Addis Ababa also reported opportunistic self-referrals to the private sector by public sector health workers; however, patients reported eventually getting the service in the private sector at a higher price [12]. In contrast, patients in this study were redirected back to the public facility without getting the service in the private clinic. The motive for this is unclear, and demands further study. This finding needs special attention and intervention from the government in order to minimize predatory behaviors such as patient diversion and demand inducement.

### Limitations of the study

This study had some limitations and potential sources of bias. The physicians’ survey was self-administered, so respondents may have been reluctant to discuss any negative effects of dual practice. Also, patient participants may have been influenced by fear of physicians who were treating them. Sophisticated analyses were not also made to control the potential source of bias.

## Conclusions and recommendations

Circular diversion pattern of referral system is the key negative consequence of dual practice and dual practitioner used public hospital to establish connection and have become gate-keepers for accessing them. Physicians and hospital managers agreed that health worker retention was the main positive consequence of dual practice upon the public sector, and banning dual practice would result in a major loss of senior physicians that would result in compromising quality of care in the public sector. The motive behind the circular diversion pattern described by patients should be studied further.

## Abbreviations

ART: Antiretroviral treatment
CEO: Chief executive officer
DOTS: Direct Observation and treatment
DP: Dual practice
EtB: Ethiopian birr
FGD: Focus group discussion
GP: General practitioner
HCT: HIV Counseling and testing
OPD: Out patient department
PMTCT: Prevention of mother to child transmission
TNRS: Tigray national regional state
WHO: World Health Organization
