# Correction to: Public-on-private dual practice among physicians in public hospitals of Tigray National Regional State, North Ethiopia: perspectives of physicians, patients and managers

**DOI:** 10.1186/s12913-017-2773-3

**Published:** 2017-12-05

**Authors:** Goitom Gigar Abera, Yibeltal Kiflie Alemayehu, Jeph Herrin

**Affiliations:** 1 Tigray Regional Health Bureau, Mekelle, Tigray Ethiopia; 2 Department of Health Services Management, College of Public Health and Medical Sciences, Jimma University, Jimma, Oromia Ethiopia; 3 Yale University School of Public Health, New Haven, CT USA

## Correction

After the publication of this article [1] it has come to our attention that the author Jeph Herrin was incorrectly included as Jeph Henry. The correct spelling is included in this erratum and the original article has been updated.
